# Factors associated with knowledge, confidence, self-efficacy, and satisfaction in African American men’s decisions about prostate cancer screening

**Published:** 2025-01-30

**Authors:** Margarita Echeverri, Kyazia Felder, David Anderson, Elora Apantaku

**Affiliations:** 1 Division of Clinical and Administrative Sciences, College of Pharmacy, Xavier University of Louisiana, New Orleans, LA 70125, USA; 2 Department of Mathematics, College of Arts and Science, Xavier University of Louisiana, New Orleans, LA 70125, USA; 3 Department of Family and Community Medicine, School of Medicine, Tulane University, New Orleans, LA 70112, USA

**Keywords:** Prostate cancer, Prostate-specific antigen (PSA), PSA screening, African American men, Baseline assessment, Confidence, satisfaction and efficacy

## Abstract

**Background:**

African American men (AAM) have persistently had the highest incidence and mortality rates for prostate cancer (PrCa) in the United States. Considering that current guidelines recommend the practice of shared decision-making to determine whether or not to undergo a Prostate-Specific Antigen (PSA) test for the early detection of PrCa, this study focuses on the identification of key factors influencing AAM decisions regarding having or not having PSA screenings.

**Methods:**

Cross-sectional study of 152 AAM who completed study surveys. Statistical analysis included frequencies, means, and distributions and methods to test for differences in knowledge confidence, satisfaction and, self-efficacy when making decisions.

**Results:**

94% of participants would get a PSA test if offered, only 57% knew that the PSA test is a blood test. Participants who reported having had a PSA test before the baseline had significantly higher mean scores than their counterparts in the knowledge about the definition of the PSA and biopsy exams (*p* = 0.04), and in the confidence (*p* = 0.005) and efficacy (*p* = 0.002) scales when making PSA screening decisions. Older participants were more likely to have had a PSA test (*p* < 0.0001) and to intend to screen (*p* = 0.0441).

**Conclusions:**

Significant differences in the satisfaction scale by clinical site (*p* < 0.001) may underscore the influence of clinicians’ practices in participants’ satisfaction with their decisions. Results suggest that patients’ experience of care has the potential to positively influence PSA screening. It is our call that type of health insurance, knowledge about PrCa and PSA, and having had a PSA test in the past, as well as the patient’s characteristics (age, race and family history of PrCa) be considered when discussing with patients the harms/benefits of PSA screening and their preferences to have or not have the PSA test.

## 1. Introduction

The U.S. Preventive Services Task Force (USPSTF) was created in 1984 by the Congressional Mandate to produce evidence-based recommendations for best practices in clinical preventive services, including the prostate-specific antigen (PSA)-based screening for early detection of prostate cancer (PrCa) [1]. In 2018, instead of recommending PSA tests for specific populations, the USPSTF recommended selectively offering or providing screening for the early detection of prostate cancer to patients, based on clinicians’ judgment and patient preferences, and called for shared decision-making about the benefits and harms of PSA-based screening between clinicians and all men ages of 55 and 69 years old, and especially between clinicians and men among high-risk groups, such as African American men (AAM) and men with a family history of prostate cancer [2]. Specifically, the guidelines recommend that men be adequately informed and educated about prostate cancer risks and screening, discuss with their healthcare providers the benefits and limitations of PSA testing, and make an informed personal choice, based on the patient’s own values and preferences, about whether to be screened. Given that the USPSTF guidelines state that “PSA-based screening is the usual method of screening” and recognize that evidence is insufficient to support defining a PSA value threshold to assess risk or using other PSA values, like free PSA levels or PSA velocity, for the stratification of individuals risks [2], current studies are recommending the incorporation of risk prediction in the screening guidelines [3, 4]. Shared decision-making (SDM) is considered a standard of person-centered care in clinical practice [5]. Educational interventions fostering SDM focus on developing decisional aids and assessments that are able to positively impact the quality of clinical services and patient-clinician communications, which may increase participants’ trust and satisfaction with the healthcare system.

According to the American Cancer Society, “Black men in the U.S. and Caribbean have the highest documented prostate cancer incidence rates in the world” [6] (pp. 24). Although for the years 2015–2019 prostate cancer has had the highest cancer incidence rates (109.9 per 100,000 male population) for all men in U.S., the incidence of prostate cancer is more than 70% higher in non-Hispanic Black men than in non-Hispanic White men [6]. Changes in prostate cancer incidence rates are largely influenced by changes in screening with the prostate-specific antigen (PSA) blood test [6] (pp. 23). Although, in the United States, prostate cancer death rates have decreased in the last few decades (from 39.3 in 1993 to 18.8 in 2020 per 100,000 male population), it is estimated that all men have a 12.6% probability (1 in 8) to develop invasive prostate cancer at some point in their lives [4]. For the year 2022 specifically, it was estimated that non-Hispanic Black men would be more likely to develop prostate cancer than non-Hispanic White men (183.4 *vs.* 110.0 cases per 100,000 men) and would be more than twice as likely as non-Hispanic White men to die of prostate cancer (37.5 *vs.* 17.8 deaths per 100,000 men) [7]. This higher death rate is attributable in part to an earlier age at cancer onset, more advanced cancer stage at diagnosis, and higher rates of more aggressive prostate cancers (*i.e.*, higher tumor grade). These differences may indicate that, in general, African American men may have lower access to high-quality care. Based on these disparities, and recognizing the epidemiologic data showing that African American men may develop prostate cancer at younger ages, the USPSTF recommends that clinicians inform African American men about their increased risk of developing and dying from prostate cancer, as well as the potential benefits and harms of screening, so they can make an informed, personal decision about whether to be screened, even at younger ages. This recommendation was backed by the Prostate Cancer Foundation’s new screening guidelines for Black men in the U.S. [8].

Considering the higher rates of prostate cancer in AAM, recent studies have focused on identifying predictors of prostate cancer screening among African American men. A study among 447 AAM attending PrCa health events identified the main predictors of undergoing routine PrCa screening as being of old age; knowledge about prostate cancer; awareness of family history of PrCa; and having a primary care provider(PCP) [9]. Another study among 174 AAM identified that self-efficacy and perceived risk of developing PrCa were significant predictors of intention to have a PrCa screening [10]. An additional study among 65 AAM found that knowledge of prostate cancer was positively associated with receipt of a PSA test [11]. Considering that these studies focused only on a few factors influencing PrCa screening, the aim of this study is to identify key factors contributing to African American men’s knowledge about prostate cancer screening and risks, as well as their confidence, self-efficacy, and overall satisfaction when making decisions regarding whether or not to have a PSA screening test. Based on the literature review, our hypothesis is that participants with a family history of PrCa and those who have had previous PSA screenings will have higher scores in knowledge, confidence, self-efficacy, and satisfaction than their counterparts. It is our intent to contribute to the discussion about the need to consider individual patient characteristics when offering patients support when making decisions about PSA screening during their medical encounters [12].

## 2. Materials and methods

### 2.1 Study design, setting and participants

Cross sectional study of African American men receiving primary care services at two clinical sites, Tulane Medical Center (TMC) and the University Medical Center (UMC), in New Orleans, Louisiana. The majority of the population in Orleans Parish, where New Orleans is located, is Black (56%), and these two clinical sites were selected because they are located in the central area of New Orleans and serve different populations. TMC primary clinics are scattered throughout New Orleans in more upscale communities and the suburbs; while they do accept Medicaid, they are not located in the lower-income areas of the city. On the other hand, UMC was opened in 2015 and focused on filling in the gaps in service to marginalized or at-risk communities. It replaced the region’s former safety net hospital, Charity Hospital, after its closure due to damage from Hurricane Katrina in 2005 and remains the region’s only safety net hospital.

After clinics’ Institutional Review Board (IRB) approvals, eligible participants were identified through a search of electronic medical records, using the following inclusion criteria: (1) self-identified as being a male of African American or Black heritage; (2) being 40 to 69 years old when enrolling in the study; (3) being active patients receiving primary care services, during the study timeline, in a study clinical site; and (4) being able to read and understand spoken English (5th grade level). Eligible participants were excluded if they: (1) had a diagnosis or personal history of prostate cancer (ICD-10-CM codes C61 or Z85.46, respectively) at the time of enrollment; (2) had medical conditions that inhibited them from completing any aspect of the study; or (3) were not willing to give signed consent. A total of 200 randomly selected eligible patients (100 at TMC and 100 at UMC) were invited to participate via emails and phone calls. Those who enrolled and signed the consent answered demographic questions and completed baseline surveys. Surveys were completed online or in-person, according to participants’ needs and preferences. Data was collected from June 2020–December 2022 using the Qualtrics XM Platform (Version 2020–2023, Qualtrics, Provo, UT, USA), and participants received a $40 electronic gift card as appreciation for their time.

Although the USPSTF guidelines recommend PSA screening for early detection of prostate cancer only for men 55–69 years old, in consideration of the USPSTF’s recommendation of PSA screening at earlier ages for African American men and men with a family history of prostate cancer [2], our sample included participants 40–69 years old.

### 2.2 Measures and instruments

#### 2.2.1 Demographic survey

Data collected included age (years old, at enrollment); education (1 = Less than high school, 2 = High school/General Educational Development (GED) degree; 3 = Some college but no degree; 4 = Associate, vocational or technical degree; 5 = Bachelor’s degree; and 6 = Advanced studies); occupation (1 = Employed full-time; 2 = Employed part-time; 3 = Self-employed; 4 = Homemaker; 5 = Student; 6 = Retired; 7 = Disabled; and 8 = Unemployed); health insurance (having or not having health insurance); history of PSA screening (having had or not having had a PSA test before enrolling to the study); prostate cancer family history (having or not having first-degree relatives with prostate cancer); and intention to receive or decline the PSA test if offered. Additionally, participants answered questions aimed to self-report their health status (1 = Poor; 2 = Fair; 3 = Good; and 4 = Very Good/Excellent), financial status (1 = Difficult; 2 = Getting by; 3 = Doing Okay; and 4 = Comfortable) using items from the Survey of Household Economics and Decision-Making [13], and health literacy level using the four items in the BRIEF Health Literacy Screening Tool [14]. Considering that type of health insurance can have an impact on the coverage of a PSA test, we asked participants who have health insurance to indicate the type of insurance (1 = Medicare, 2 = Medicaid, 3 = Insurance purchased directly from an insurance company or through an exchange or marketplace, 4 = Insurance through a current or former employer or union, and 5 = Other types of insurance such as the ones provided by the USA Veterans Administration (VA) and the USA Uniformed Services (TRICARE)).

#### 2.2.2 Baseline surveys

Participants completed six instruments measuring knowledge about PrCa and PSA screening, as well as confidence, satisfaction and decisional self-efficacy when making health-related decisions (Table 1, Ref. [15–18]). These instruments were selected after an evaluation of the most common cancer screening tools and decisional scales found in the literature and were adapted, as needed, to be specifically targeted to the topic of interest: PSA screenings for the early detection of prostate cancer. Once adapted, the instruments were evaluated and revised by the African American Patient Advisory Board (AAPAB) and pilot tested with 12 individuals. The AAPAB is an Advisory Board that has collaborated with the investigators for more than seven years in cancer-related studies targeted toward African American communities in general. For this study, the AAPAB consisted of eight African American members (six men and two women). Two of the men were prostate cancer survivors, two men had a family history of PrCa, and the two women had first-grade relatives who had or died of PrCa. In order to accommodate for differences in age and literacy level of study participants, the AAPAB members and participants in the pilot test included individuals between three age ranges (40–49, 50–59, 60–69) and different levels of education and occupation.

Knowledge scales included three measures that were developed based on the Prostate Cancer Screening (PROCASE) questionnaire [15]. Questions were classified into three subscales according to the topic. Knowledge 1 (K1) measured participants’ knowledge about symptoms and risk of having or dying from prostate cancer. Knowledge 2 (K2) measured participants’ understanding of the definition of PSA tests and biopsy exams. Knowledge 3 (K3) measured participants’ knowledge about PrCa screening, reliability of PSA results, and possible side effects of the prostate biopsy and prostate cancer surgery along the life span.

Decision scales included three measures. Confidence measured participant certainty when making the decision about PSA screening, including understanding the options available, possible benefits and harms of each option, one’s values and preferences, as well as the presence of enough information and support needed to make the decision [16]. Satisfaction measured participant level of satisfaction with the decisions they have made so far, including readiness to make the decision and consistency of decisions made with personal preferences [17]. Decisional Self-efficacy measured participant capability to ask questions and get the information needed to make their own decisions and understand the impact of their decisions in their lives [18].

### 2.3 Statistical analysis

After initial exploratory analyses, the demographic variables education, occupation, and type of health insurance were condensed. Education categories were condensed into three groups: 1 = less than high school, 2 = GED or high school diploma, and 3 = more than high school diploma. For occupation, the category “other” was created to group participants who reported being part-time employed (n = 12); self-employed (n = 5); and unemployed (n = 9). Similarly, insurance was condensed into three categories (0 = none; 1 = Public insurance; and 3 = Private insurance). Finally, considering that, in general, (1) guidelines recommend PSA screening only for men 55–69 years old, (2) most men 50–59 years old have rutinary PSA screenings, and (3) for AAM, some guidelines specifically recommend screening at younger ages, participants were, intentionally, grouped according to their age into three age-range groups (40–49, 50–59 and 60–69).

Response (dependent) variables were knowledge scores (K1, K2 and K3); confidence (CO), satisfaction (SA) and self-efficacy (SE) scores; whether the participant had received PSA test screening before enrollment (History-Having-PSA-Tests), and whether the participant would like to have a PSA screening (Intention-to-Screen), if offered. For each response variable, the following explanatory (independent) variables were considered: age; education; occupational status; financial status; self-reported health status; health literacy score; insurance, the clinic where the participant received primary care services; whether the participant had a family history of prostate cancer (PrCa-Family-History); and whether the participant knew that the PSA test is a blood test, instead of a digital rectal exam (DRE), for early detection of prostate cancer (Knew-PSA-blood-test).

Numerical response variables were analyzed using ANOVA (Analysis of Variance), while binary response variables were analyzed using logistic regression. A squared transformation was used on satisfaction scores, and a log transformation was used on self-efficacy scores to solve diagnostic problems. The self-efficacy results were also checked with a nonparametric Kruskal test. A Holm correction was used for multiple comparisons. In accordance with standard statistical practice, explanatory variables were tested for significance after controlling for all other significant explanatory variables [19].

Statistical packages SPSS (version 23; SPSS Inc., Chicago, IL, USA, RID: SCR_002865) and R were used to carry out the data analyses.

## 3. Results

### 3.1 Demographics of participants

A total of 152 participants, out of the 200 AAM invited to join the study (Response Rate = 76%), completed the study questionnaires. The majority of participants were 50–69 years old; participants at the Tulane Medical Center (TMC); did not have a family history of PrCa; had had a PSA screening before enrollment; knew that the PSA test is a blood test; and would like to be screened, if offered (Table 2). While similar percentages of participants had less than high school education (23%) or at least a high school diploma (29%), a higher percentage of participants (48%) had been exposed to some level of higher education, categorized by having some college studies and/or having undergraduate or advanced degrees. Regardless of educational attainment, the majority of participants (n = 97, 64%) self-rated their health literacy level in the highest level (17 to 20, range 4–20).

Significant differences (*p* < 0.001) by clinic were found in education, occupation, and health insurance (Table 2). While higher percentages of participants in TMC had advanced education, were full-time employees, and had private insurance, higher percentages of participants at UMC did not have a high school diploma, were disabled, and had public health insurance.

### 3.2 Key factors influencing PSA decision

#### 3.2.1 Type of health insurance

Although the majority of participants in the study had health insurance (n = 143, 93%), most of the participants in TMC had private insurance through an employer or marketplace (n = 45, 54%), while most of the participants in UMC (n = 58, 85%) had public insurance (Medicaid, Medicare, VA, TRICARE, *etc.*). Significant differences by clinical site in type of health insurance (*p* < 0.001) and education (*p* < 0.001) were found. Specifically, the majority of participants with both a higher education level and private insurance (n = 36) go to TMC (n = 33, 92%), while the majority of participants with both a low educational level and public insurance (n = 31) go to UMC (n = 24, 73%). No significant differences were found by health insurance in the history of receiving a PSA or intention to screen.

#### 3.2.2 History of receiving a PSA (History-PSA-Tests)

Less than half (n = 68, 45%) of participants reported that they had never had a PSA screening (Table 2). Among those who reported that they had had a PSA test before baseline (n = 84, 55%), a higher percentage of participants at the TMC site (n = 51, 61%) reported having had a prior PSA test than those at the UMC site (n = 33, 49%). Although clinical site did not have a significant effect in having had a PSA test before enrollment (adjusted *p*-value = 0.09), older participants were significantly more likely to have had a PSA test (*p* < 0.0001, OR (Odds Ratio) = 1.13 for each additional year of age, 95% CI (confidence interval) [1.06, 1.17]).

#### 3.2.3 Intention to screen (intent-to-screen)

Regardless of having had or not having had a previous PSA screening, most participants (n = 144, 95%) would get a PSA screening, if offered (Table 2). However, older participants were more likely to intend to screen (*p* = 0.0441, OR = 1.11 for each year older, 95% CI [1.003, 1.23]).

#### 3.2.4 Knowledge about PSA (Knew-PSA-blood-test)

The majority of participants knew that the PSA test is a blood test (n = 86, 57%). Participants who wrongly believed that the PSA test is not a blood test (n = 15, 10%), and those who did not know what type of test the PSA test was (n = 51, 34%), were grouped for the analysis (Table 2). Participants who correctly answered that the PSA test was a blood test to screen for prostate cancer were more likely to self-report that they have had a PSA test (*p* < 0.0001, OR = 5.06, 95% CI [2.38, 10.73]) than their counterparts.

In summary, while age and knowledge about the type of test the PSA test is were significantly related to having had a PSA test and intention to have one (Table 3), the type of health insurance and having a family history of prostate cancer did not have a significant effect in these decisions.

### 3.3 Mean scale scores

In general, significant differences in mean scale scores were found by knowing that the PSA test is a blood test; having received previous PSA tests; family history of PrCa; insurance type; intention to screen; education; age; and clinical site (Table 4). However, no significant differences were found in any of the mean scale scores by literacy level, occupation, financial status, and health status.

Significant differences in mean scale scores by different demographic characteristics (explanatory variables) are explained below:

- Knowledge 1 (K1) scores: Significantly higher K1 mean scores were reported by participants who know the definition of the PSA test, have a PrCa-family-history, and would receive a PSA screening test, if offered (Table 4). As there were no participants who had a family history of PrCa but did not intend to screen, a combined effect of these two variables also had a statistically significant impact on K1 (*p* < 0.001). Participants who had PrCa-family-history and intended to screen had a higher K1 score than other participants (*p* = 0.020). Additionally, participants with private insurance had significantly higher K1 scores than those with public insurance. However, there were too few participants with no insurance (n = 9) to find significant differences for those participants.
- Knowledge 2 (K2) scores: K2 scores were significantly impacted by whether participants Knew-PSA-blood-test, had a history of receiving the PSA test, and education level (Table 4). At a 95% confidence interval, K2 scores were between (0.01, 0.66) points higher for participants with a History-Having-PSA-Tests, and between (0.99, 1.64) points higher for participants who Knew-PSA-blood-test. Additionally, education level had a statistically significant impact on K2 and a large effect size (Cohen’s *F* = 0.43, 95% CI for Cohen’s *F* [0.25, 0.59]). Specifically, K2 scores were significantly higher (*p* value < 0.001) for participants who had more than a high school education than for participants with only a high school diploma or lower educational level.
- Knowledge 3 (K3) scores: For K3 scores, there was a statistically significant impact for Knew-PSA-blood-test, PrCa-family-history, and age-range. Significantly higher K3 scores were found for participants who Knew-PSA-blood-test (*p* < 0.001, 95% CI [0.49, 1.64]) and those participants with PrCa-family-history (*p* = 0.016, 95% CI [0.16, 1.55]). There was also a statistically significant interaction between age-range and Knew-PSA-blood-test (*p* = 0.0076). Specifically, participants who were both in the 40–49 age range and did not Knew-PSA-blood-test have lower K3 scores than all other groups (*p* < 0.026).
- Confidence scores: Confidence scores were significantly higher for participants with a History-Having-PSA-Tests (*p* = 0.005, 95% CI [1.21, 6.65]) and for participants with insurance (*p* = 0.045, 95% CI [0.14, 11.57]). Confidence scores were also significantly higher (*p* = 0.045) for participants who had health insurance, regardless of insurance type, than those without health insurance.
- Satisfaction scores: Satisfaction scores were significantly higher for participants who Knew-PSA-blood-test (*p* = 0.007) and participants at the UMC clinic (*p* < 0.001).
- Self-efficacy scores: Self-efficacy scale scores were significantly higher for participants with a History-Having-PSA-Tests (*p* = 0.002). Insurance type also had a significant impact on self-efficacy scores (*p* = 0.027). Specifically, participants with private insurance had significantly higher self-efficacy scores than participants with public insurance (*p* = 0.0032). However, there were too few participants with no insurance (n = 9) to find significant differences in self-efficacy scores for those participants.

In summary, compared to their counterparts, participants who knew that the PSA test was a blood test had significantly higher K1, K2, K3, and satisfaction scores; participants who reported a history of receiving the PSA test had significantly higher K2, confidence, and self-efficacy scores; and participants who had a family history of prostate cancer reported higher K1 and K3 scores. Additionally, participants who had the intention to screen reported higher K1 scores and participants at UMC reported higher satisfaction scores than their respective counterparts.

## 4. Discussion

In this cross-sectional study of AAM participants (40–69 years old) who completed demographic and baseline surveys, eight factors that may influence their knowledge about PrCa and their confidence, satisfaction, and self-efficacy when making decisions regarding having or not having a PSA screening test were identified: knowledge that PSA is a blood test; having had a PSA test in the past; having a family history of PrCa; type of health insurance; intention to screen; education; age; and clinical site. The influence of these factors on the participants’ knowledge, confidence, satisfaction and self-efficacy when making decisions is discussed below.

### 4.1 Family history of PrCa and knowledge regarding the PSA test

Our results showed that participants with a family history of PrCa had significantly higher scores in K1, K3, and satisfaction with their decisions. Additionally, understanding that the PSA test is a blood test, instead of a digital rectal exam, was one of the key factors influencing participants’ knowledge of prostate-cancer issues (K1, K2 and K3) as well as satisfaction with their own decisions. Furthermore, our results found a statistically significant relationship between participants’ understanding that the PSA is a blood test and having a PSA test (*p* < 0.001). Several studies have reported that men with a strong family history of PrCa have a greater awareness of prostate cancer and look for more early screening and diagnosis opportunities. Specifically, a study about European men with prostate cancer, and who also have a first-degree or second-degree relative with prostate cancer, found that these participants were 15% more likely to survive prostate cancer because of their heightened awareness of cancer risk and being more proactive getting screened than those with no family history of PrCa [20]. A study about Black men eligible for PSA screening found that those with a family history of PrCa were more motivated to seek screening [21]. Unfortunately, family risk has been overlooked by men and their clinicians; while half of all UK men do not know if they have a PrCa family history, two-thirds of men in the UK with a family history of prostate cancer are unaware of their increased risk [22]. Another study using data from the National Health Interview Survey (NHIS) in a sample of 1744 men, more than 40 years old, who reported having “ever heard of a PSA test”, found that those men with a family history of PrCa were more likely to have a PSA test, however, when controlling by race, Black men with a PrCa family history were not significantly more likely to have a PSA test than their counterparts [23].

Although our study targets only African American men, who are at a higher risk of PrCa, the majority of our participants (n = 121, 83%) reported that they have no family history of PrCa. Unfortunately, this information was self-reported, so it is possible that in our study, some of these participants may not be aware of their own family history. A recent study reported that less than one-third of the general population in the US knew of their family history information [24], and another study, among 447 African American men, found that 27% did not know if they had a family history of prostate cancer [9]. Focus groups and interviews with African American communities talking about their family cancer history identified five key barriers to sharing family cancer history (fear/denial; pride/dignity; selflessness/self-sacrifice; cancer fatalism; and distrust/skepticism of medical care) and concluded that, although most participants had experienced cancer in their families, communication about family cancer history was low [21].

### 4.2 Type of health insurance and confidence

Participants’ confidence when making decisions regarding having or not having a PSA screening test was influenced by their financial status (crude *p* = 0.021). However, financial status no longer had a statistically significant impact after controlling for whether or not participants had health insurance. We found that participants with health insurance had higher confidence scores (adjusted *p* value = 0.045). Considering that Medicare Part B and Medicare Advantage plans cover yearly PSA screening tests (with no co-insurance and deductible) for men over 50 years old and that many states have laws requiring private health insurance to also cover the PSA screening test for high-risk men (African Americans and those with family history of PrCa), who are 40+ years old [25, 26], it makes sense that having or not having health insurance, instead of type of health insurance (private *vs.* public), had an impact on confidence scores. Although, in our study, there were no significant differences in insurance type and history of receiving PSA tests before enrollment (*p* = 0.158), a recent study reporting PSA testing practices in an academic health organization found that participants with private insurance or a mix of private/public plans were significantly more likely (*p* = 0.014) than participants with public insurance or no insurance to have a PSA test [27].

Although we were not able to find evidence about the possible connection between health insurance status and confidence in health decision-making, a 2020 study, focused on the performance of 167 individuals conducting laboratory experiments while they had access to different types of insurance or no insurance at all, found that individuals with appropriate insurance coverage for their perceived risk, were more confident when making own decisions along their daily activities [28].

### 4.3 Clinical site and satisfaction

Results show that participants in the TMC site had significantly lower mean scores (M) on the satisfaction scale(M = 21.5) than participants in the UMC site (M = 25.0). Although the majority of participants in the TMC clinical site had a higher education level, private insurance, and higher confidence scores than those in the UMC site (Tables 2 and 3), important differences between TMC and UMC may influence clinicians’ practices when discussing PrCa screening. While TMC has been part of Tulane University, a prestigious private university, located in the higher-income areas of the city, UMC is perceived as the new “Charity Hospital” that provides services to a high percentage of minority and marginalized patients covered by Medicaid and Medicare [29].

A retrospective study evaluating the impact of men’s perceptions of healthcare quality on obtaining a PSA screening for early detection of PrCa found that men who rated the quality of healthcare delivered to them as high (≥7, range 0–10) had significantly greater odds of undergoing PSA screening compared to those who rated it lower [30]. Additionally, two other studies found that physicians’ recommendations and decisions in ordering a PSA test were among the more influential factors in screening [31, 32]. During the study, it was observed that TMC offers free monthly prostate cancer screenings [33, 34] and that some TMC clinicians routinely ordered the PSA test before a medical encounter, so, during the medical encounter, they focused the conversation on the results of the pre-ordered test, instead of the discussion about participants’ risks, values, and preferences of being screened, which may hamper the application of the SDM process during the medical encounter. In a study among 276 Black men with no diagnosis of prostate cancer, it was found that “men who utilized health systems with a prostate cancer screening policy had high percentages of PSA and DRE (63.3%), PSA only (70.9%) and DRE only (81.7%)” and concluded that physicians who aggressively promote prostate cancer prevention among Black men, combined with institutional screening policy, highly increase early detection within this population group [12].

### 4.4 Shared decision-making and intention-to-screen

As explained earlier, the USPSTF guidelines for PSA-based screening for early detection of PrCa recommend that male patients and their providers engage in a shared decision-making (SDM) process about the benefits and harms of PSA-based screening [2]. Through shared decision-making, primary care providers (PCPs) can empower patients to understand their personal risk and their perceptions of the benefits, harms, and uncertainty of PSA-based screening in order to make an individualized decision as to whether screening is right for them. These conversations are especially important for African American men, given increased ambiguity due to the lack of PSA-based research specific to this population and increased risk of prostate cancer mortality attributed to late-stage diagnoses and more aggressive prostate cancer phenotypes seen in African American men.

Instead of encouraging an increase in PSA-based screening (expected positive behavioral change), the guidelines make PSA-based screening a shared decision, whether patient preferences for or against receiving PSA-based screening should align with care received instead of being driven by behavioral change theories or practice-level default norms. Application of SDM may have an important impact in increasing quality of healthcare services provided (higher satisfaction and quality of decisions implies higher patient satisfaction with services) and increasing access to and utilization of services that patients prefer, thereby improving the overall quality of life and addressing health disparities outcomes.

In this study, the majority of participants (n = 144, 95%) intent to screen, regardless of the recommendations they may have or not received from their healthcare providers. Although SDM and behavioral change interventions were outside of the scope of this study, a “successful” application of shared decision-making would be “preference-concordant” if patients opting for PSA-based screening actually receive it, and if patients who do not want PSA-based screening avoid receiving it. In the other direction, “preference incongruence” would indicate a mismatch between intention-to-screen and real action.

### 4.5 Limitations

Limitations of this study are discussed below.

First, as data collection was through self-reported surveys, participants’ responses may be influenced by external factors. In demographic, epidemiological, and medical research, it is common to collect “self-reported” data through surveys or questionnaires where participants answer questions directly by themselves and without the influence of the researcher. Depending on the question, participants use their memory (recall) or personal perceptions (agreement *vs.* disagreement) to answer the question. In this study, it may be possible that some of the participants did not accurately remember if they had a PSA test before, or may not be aware of their own prostate cancer family history (recall bias). It may also be possible that participants answered questions in the decision surveys (confidence, satisfaction, and self-efficacy) in the positive range (high scores) because they wanted to show that they were doing well (social desirability bias), instead of answering the questions truthfully [35]. In order to reduce survey response bias, participants completed the survey online, with no time restrictions. They were also instructed to respond truthfully, and were assured that the information was anonymous and confidential.

Second, as the study focused only on African American men receiving care in two clinical sites (TMC and UMC) in the Greater New Orleans Area, results may not generalize to patients with different racial backgrounds, different socioeconomic status, living outside this geographic area, or receiving primary care in other healthcare systems. This study was limited to African American men because of the high prostate cancer risk in this population group, and the two clinical sites were chosen because of the different characteristics of the patient population in each site. It is recommended to conduct similar studies in different population groups, regions, and healthcare systems.

Third, because of the small sample size, some important relationships may have been missed, and this study is not powered to draw conclusions regarding predictors of PSA screening or to permit generalization of results to a wider population. Because of these limitations, results should be interpreted with caution.

Fourth, as this study used across-sectional design and results were limited to data collected at a single point in time, it is not possible to establish causal relationships between the variables of interest.

Fifth, although this study examined age; education; health literacy level; occupation; financial status; health status; health insurance; and family history of PrCa as possible confounding factors influencing confidence, satisfaction and self-efficacy of participants when making decisions about PSA screening, other factors such as weight, marital and smoking status, as well as medical conditions (such as benign prostatic hyperplasia and prostatitis), and medications (such as NSAIDS) affecting prostate health, may also influence participants’ decisions to whether or not to have a PSA test.

Lastly, considering that the focus of this study is to identify key factors contributing to patient decisions regarding having or not having a PSA test, consideration of risk prediction methods for prostate cancer is outside of the scope of this study.

## 5. Conclusions

This study focused on factors associated with knowledge, confidence, self-efficacy and satisfaction in African American Men’s decisions about prostate cancer screening. Regardless of the limitations, this study found three key variables that have a significant impact in participants’ knowledge regarding PrCa and screening as well as in their confidence, satisfaction, and self-efficacy when making decisions about whether to be screened or not: (1) having a family history of PrCa; (2) having had previous PSA screenings; and (3) understanding that although the PSA test and the DRE (Digital Rectal Exam) are both tests for early detection of prostate cancer, the PSA test is a blood test that should not be confused with the rectal examination. In particular, knowing that the PSA test is a blood test had large to moderate effect sizes for all six mean scales. Although there is a need to study the influence of knowledge on confidence, satisfaction, and self-efficacy when making decisions, other factors such as education, insurance, and clinical settings also play a crucial role in these decisions.

Our results may suggest that improvements in healthcare quality and patient experience of care have the potential to positively influence PSA screening. It is our call that factors, such as type of health insurance, clinical site, knowledge about PrCa and PSA, and having had a PSA test in the past, as well as the individual patient’s characteristics (age, race, and family history of PrCa) be included when developing decision aids that are offered to participants before or during their medical encounters. More specifically, asking participants and encouraging them to know and discuss their family history of prostate cancer, as well as other genetic conditions, should be required when applying SDM and developing interventions to address disparities in cancer prevention and early detection, particularly where guidelines recommend PSA screening for those with positive family histories.

## 6. Future directions

In order to address the limitations of this study and advance the research topic, directions for future studies include: (1) conducting research using participants’ data from medical records to confirm reports about their family history and actual PSA screenings (addressing recall bias); (2) including PSA test results to consider the possible impact of risk prediction in having prostate cancer; (3) including other racial/ethnic groups, regardless of their risk of prostate cancer, so it is possible to identify potential confounding factors that may influence their willingness to have a PSA screening test; (4) use a longitudinal cohort design, including additional confounding variables, to better understand factors influencing changes in decisions, confidence, satisfaction and self-efficacy over time; (5) focus on the development and/or assessment of interventions using the shared decision-making process to better understand the complex interactions of the underlying mechanisms that influence decision-making and behavior change regarding PrCa screening for early detection; and (6) extend the study to other clinical sites and, perhaps, other cancer screenings, such as colonoscopies and mammograms, to identify if the type of clinical site or screening test may have a confounding influence on the variables of interest.

## Figures and Tables

**TABLE 1.** Baseline study measures.

Scales^1^	Number of Items	Rating	Range^2^
PrCa Knowledge scales [15]			
Knowledge 1 (K1): Prostate cancer risks/facts	7	1 point for each correct answer	0 to 7
Knowledge 2 (K2): Definition PSA and biopsy	4		0 to 4
Knowledge 3 (K3): Screening, diagnosis, and treatment of prostate cancer	9		0 to 9
PSA Decision scales			
		4 points for each “YES”	
Confidence in decision making [16]	10	0 points for each “NO”	0 to 40
		2 points for each “NO SURE”	
Satisfaction with decisions made [17]	6	Likert Scale, 1 to 5	6 to 30
Decisional Self-efficacy [18]	4	Likert Scale, 1 to 5	to 20

1 Measures used in this study have been adapted from the ones found in the literature.

2 Scores are directly related to the respective scales. For example: Higher scores in the knowledge and decision scales mean higher quality of shared decision-making process.

PSA: Prostate Specific Antigen; PrCa: Prostate cancer.

**TABLE 2.** Demographics of African American men participants by treatment group (n = 152).

Characteristic	Total		TMC		UMC		Adjusted *p* value^a^
	n	%	n	%	n	%	
	152	100.0	84	55.3	68	44.7	
Age range							
40–49	28	18.4	19	22.6	9	13.2	0.553
50–59	56	36.8	24	28.6	32	47.1	
60–69	68	44.7	41	48.8	27	39.7	
Education							
Less than High School	35	23.0	10	11.9	25	36.8	<0.001
GED or High School Diploma	44	28.9	22	26.2	22	32.4	
More than High School^b^	73	48.0	52	61.9	21	30.9	
Health Literacy Level							
Low level (4 to 12)	22	14.5	8	9.5	14	20.6	1.000
Moderate level (13 to 16)	33	21.7	18	21.4	15	22.1	
High level (17 to 20)	97	63.8	58	69.0	39	57.4	
Occupation							
Full time employed	46	30.3	38	45.2	8	11.8	<0.001
Retired	33	21.7	17	20.2	16	23.5	
Disable	47	30.9	16	19.0	31	45.6	
Other^c^	26	17.1	13	15.5	13	19.1	
Financial Status							
Difficult	16	10.5	5	6.0	11	16.2	0.553
Getting By	55	36.2	27	32.1	28	41.2	
Doing Okay	57	37.5	35	41.7	22	32.4	
Comfortable	24	15.8	17	20.2	7	10.3	
Health Status							
Poor	16	10.5	5	6.0	11	16.2	0.218
Fair	69	45.4	34	40.5	35	51.5	
Good	52	34.2	33	39.3	19	27.9	
Very good	15	9.9	12	14.3	3	4.4	
Health Insurance Type							
None	9	5.9	4	4.8	5	7.4	<0.001
Public (Medicare, Medicaid, VA, *etc*.)	93	61.2	35	41.7	58	85.3	
Private (Employer/marketplace)	50	32.9	45	53.6	5	7.4	
Have a Family History of PrCa							
No	121	79.6	69	82.1	52	76.5	1.000
Yes	31	20.4	15	17.9	16	23.5	
Is the PSA test a blood test used to screen for prostate cancer?							
No/Don’t know (Wrong)	66	43.4	35	41.7	31	45.6	1.000
Yes (Correct)	86	56.6	49	58.3	37	54.4	
Have ever had a PSA Screening							
No	68	44.7	33	39.3	35	51.5	1.000
Yes	84	55.3	51	60.7	33	48.5	
Intention to get the PSA screening, if offered							
No	8	5.3	4	4.8	4	5.9	1.000
Yes	144	94.7	80	95.2	64	94.1	

a Test for independence with Bonferroni correction.

b Some College, Undergraduate, or Advance Degrees.

c Part-time employed, self-employed, or unemployed.

PSA: Prostate Specific Antigen; PrCa: Prostate cancer; TMC: Tulane Medical Center; UMC: University Medical Center of New Orleans; GED: General Educational Development; VA: USA Veterans Administration.

**TABLE 3.** Selected Odds Ratios for significant results.

Likelihood to	Is affected by	More likely for	Odds Ratio (OR)	95% CI for OR
Have had a PSA test	Age	Each year older (age 40–69)	1.13	[1.06, 1.17]
Have had a PSA test	Knowing that PSA is a blood test	Correct Answers	5.06	[2.38, 10.73]
Intend to have a PSA screening	Age	Each year older (age 4–69)	1.11	[1.003, 1.23]

PSA: Prostate Specific Antigen; CI: Confidence Interval.

**TABLE 4.** Scales mean scores at baseline (n = 152).

Demographic characteristics of participants (n)	Scale Mean scores (SD)					
	Knowledge^1^			Confidence in decision making	Satisfaction with decision made	Self-efficacy
	K1	K2	K3			
All participants (152)	2.75 (1.267)	1.88 (1.268)	4.29 (1.897)	26.17 (8.664)	23.04 (6.352)	9.10 (4.988)
Is the PSA test a blood test used to screen for PrCa?^2^	*p* = 0.002	*p* < 0.001	*p* < 0.001		*p* = 0.007	
No/Don’t know (66)	2.36 (1.145)	0.98 (0.903)	3.70 (2.045)		21.65 (6.738)	
Yes (86)	3.05 (1.283)	2.56 (1.069)	4.74 (1.646)		24.10 (5.857)	
Have you ever had the PSA Screening?^2^		*p* = 0.044		*p* = 0.005		*p* = 0.002
No (68)		1.46 (1.177)		24.09 (8.994)		7.37 (4.270)
Yes (84)		2.21 (1.243)		27.86 (8.053)		10.50 (5.110)
Family History of PrCa^2^	*p* = 0.004		*p* = 0.016			
No (121)	2.60 (1.215)		4.13 (1.897)			
Yes (31)	3.35 (1.305)		4.90 (1.795)			
Insurance Type^3^	*p* = 0.048			*p* = 0.045^4^		*p* = 0.027
None (9)	2.44 (1.424) AB			2.44 (1.424)		3.56 (2.455) AB
Public (93)	2.56 (1.184) B			2.56 (1.184)		4.29 (1.773) B
Private (50)	3.16 (1.315) A			3.16 (1.315)		4.42 (2.021) A
Would you get the PSA screening, if offered?^2^	*p* = 0.010					
No (8)	1.63 (1.302)					
Yes (144)	2.81 (1.240)					
Education^3^		*p* < 0.001				
Less than High School (35)		1.43 (1.145) B				
GED or High School Diploma (44)		1.36 (0.967) B				
More than High School (73)		2.40 (1.288) A				
Age Range^3^			*p* = 0.046			
40 to 49 (28)			3.54 (2.202)			
50 to 59 (56)			4.54 (1.839)			
60 to 69 (68)			4.40 (1.755)			
Clinical Site^2^					*p* < 0.001	
Tulane Medical Center (84)					21.49 (7.200)	
University Medical Center (68)					24.96 (4.467)	

p-values are after taking all other significant factors into consideration.

1 K1: Knowledge about prostate cancer (PrCa) risks and facts; K2: Knowledge about definition of Prostate-Specific Antigen (PSA) test and biopsy; K3: Knowledge about PrCa screening, diagnosis & treatments.

2 Independent Samples Test (t-test for equality of means, two-sided p, equal variances not assumed).

3 One Way Analysis of Variance (ANOVA).

4 Variable considered is whether patient has insurance or not, instead of type of insurance.

Letter plots are indicated with capital letters (A and B). Shared letters indicate no statistically significant differences among the specific groups. For example: Self-efficacy mean scores for participants with no insurance or public insurance share a letter (B), so there is no significant difference between them; however public insurance and private insurance do not share a letter (B vs. A) so there is a significant difference between them.

PSA: Prostate Specific Antigen; PrCa: Prostate cancer; SD: Standard Deviation.

## Data Availability

De-identified summary of participant data and supporting materials will be made available within 6 months of study completion. Data access requests will be reviewed by the full IRBs and principal investigator(s). If approved, requestors may be required to sign a Data Access Agreement.

## ABBREVIATIONS

AAM: African American men
PrCa: prostate cancer
PSA: Prostate-Specific Antigen
USPSTF: U.S. Preventive Services Task Force
SDM: Shared decision-making
PCP: primary care provider
TMC: Tulane Medical Center
UMC: University Medical Center
IRB: Institutional Review Board
GED: General Educational Development
VA: USA Veterans Administration
TRICARE: USA Uniformed Services
AAPAB: African American Patient Advisory Board
PROCASE: Prostate Cancer Screening
CO: confidence
SA: satisfaction
SE: self-efficacy
DRE: digital rectal exam
ANOVA: Analysis of Variance
TMC: Tulane Medical Center
OR: Odds Ratio
CI: confidence interval
SD: Standard Deviation
NHIS: National Health Interview Survey
M: mean scores
